# Glatiramer acetate attenuates the activation of CD4^+^ T cells by modulating STAT1 and −3 signaling in glia

**DOI:** 10.1038/srep40484

**Published:** 2017-01-17

**Authors:** Ye-Hyeon Ahn, Sae-Bom Jeon, Chi Young Chang, Eun-Ah Goh, Sang Soo Kim, Ho Jin Kim, Jaewhan Song, Eun Jung Park

**Affiliations:** 1 Cancer Immunology Branch, National Cancer Center, Goyang, South Korea; 2 Dept.of Biochemistry, College of Life Science and Biotechnology, Yonsei University, Seoul, Korea; 3 Dept. of System Cancer Science, Graduate School of Cancer Science and Policy, Goyang, South Korea; 4 Radiation Medicine Branch, National Cancer Center, Goyang, South Korea; 5 Dept. of Neurology, National Cancer Center, Goyang, South Korea

## Abstract

Interactions between immune effector cells of the central nervous system appear to directly or indirectly influence the progress/regression of multiple sclerosis (MS). Here, we report that glial STAT1 and −3 are distinctively phosphorylated following the interaction of activated lymphocytes and glia, and this effect is significantly inhibited by glatiramer acetate (GA), a disease-modifying drug for MS. GA also reduces the activations of STAT1 and −3 by MS-associated stimuli such as IFNγ or LPS in primary glia, but not neurons. Experiments in IFNγ- and IFNγ receptor-deficient mice revealed that GA-induced inhibitions of STAT signaling are independent of IFNγ and its receptor. Interestingly, GA induces the expression levels of suppressor of cytokine signaling-1 and −3, representative negative regulators of STAT signaling in glia. We further found that GA attenuates the LPS-triggered enhancement of IL-2, a highly produced cytokine in patients with active MS, in CD4^+^ T cells co-cultured with glia, but not in CD4^+^ T cells alone. Collectively, these results provide that activation of glial STATs is an essential event in the interaction between glia and T cells, which is a possible underlying mechanism of GA action in MS. These findings provide an insight for the development of targeted therapies against MS.

Multiple sclerosis (MS) is a chronic autoimmune disorder that affects the central nervous system (CNS), and is a leading cause of neurological disability among young adults. MS is characterized by demyelination, axonal loss, inflammation, gliosis, and the appearance of numerous plaques with immune and inflammatory cells in the CNS^1^. Approximately 85% of patients have biphasic disease courses that show alternating episodes of neurological disability followed by complete or partial recovery^2^,^3^. Fifteen years after diagnosis more than 80% of patients have functional and/or cognitive limitations, and approximately half require assistance to walk^4^. Studies have strongly suggested that MS occurs when the body’s own defense system attacks the CNS, and that disease progression is closely related with immune-mediated injury to myelin and axons^5^. Efforts have focused on the understanding MS pathogenesis and the development of effective MS therapeutics. Substantial progress has been made in MS research, and therapeutic options have increased in recent years^6^,^7^. However, the exact molecular mechanisms underlying the onset and progression of MS remain largely unknown and a salient challenge.

Although the incidence of MS has increased considerably in recent decades, we still lack curative treatment for MS^8^. Currently, disease-modifying drugs (DMDs) are used to reduce the severity and frequency of disease activity. Approximately 12 such DMDs, mostly immunosuppressant and immunomodulatory drugs, have been approved for use in MS^9^,^10^. The first line medications include IFNβ, glatiramer acetate (GA), teriflunomide, and dimethyl fumarate, which are more effective in the early phase of disease development^11^. GA is a synthetic mixture of four amino acid copolymers (L-alanine, L-lysine, L-glutamic acid, and L-tyrosine). GA was originally synthesized to resemble the structure of myelin basic protein (MBP) and was expected to provoke experimental autoimmune encephalomyelitis (EAE). Unexpectedly, it was found to prevent or minimize MBP-induced EAE^12^. It was subsequently developed and approved for the treatment MS patients^13^,^14^. Studies have suggested that oral administration of GA as well as parenteral route administration is also effective suppressing EAE models in rats, mice and in primates^15^. Efforts to uncover the relevant action mechanism(s) of GA have shown that it influences immune cells, including T cells and B cells, to confer immunological and neuroprotective effects^16^,^17^,^18^. But, the overall effects and action mechanisms of GA are not fully understood, and better understanding the mechanism of how GA ameliorates MS could lead to the development of newer and better therapies for this disease.

Inflammatory events are observed in MS-related lesions at all stages of the disease, and inflammatory aggregates are likely to cause demyelination and neuronal loss in the white and grey matter of the CNS. As the disease progresses, both T cell infiltrates and activated glial cells increase markedly, and elicit immunological neurodegenerative responses^1^. The dysregulation of these inflammatory cells further recruits/activates T cells and innate immune cells. These inflammatory events appear to closely involve crosstalk among cells in the CNS^19^,^20^. Studies have strongly supported that disease progression is closely related with immune-mediated injury to myelin and axons, and that not only T-cell-mediated immune events but also activation of innate immune system may contribute to this devastating disease^5^,^21^. In this study, we reveal that glial STAT1 and -3 are potential signaling molecules in glia- and T cell-associated neuroinflammation, and that this signaling event is a possible molecular mechanism underlying the effect of GA on MS. Our results suggest that GA efficiently modulates the activations of STAT1 and −3 in glial cells, brain-resident immune cells, and that this affects the activation of T cells and the production of inflammatory mediators. These observations improve our understanding of the molecular mechanisms underlying MS and may facilitate the development of effective targeted therapies against this disease.

## Results

### Activated lymphocyte-triggered phosphorylations of glial STAT1 and −3 are significantly reduced by GA

**Figure 1:**
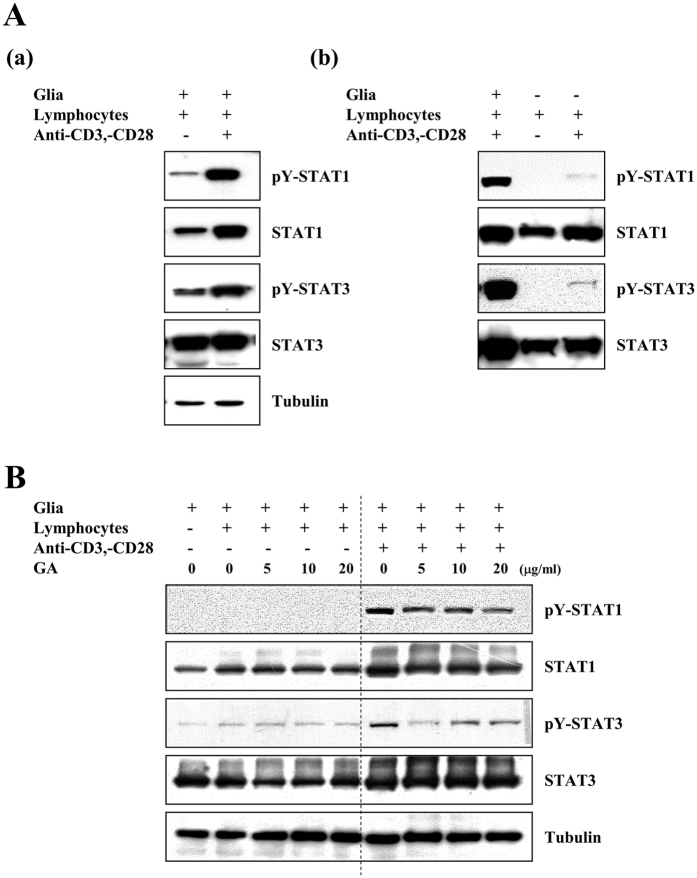
GA markedly reduces the activated lymphocytes-triggered phosphorylations of STAT1 and −3 in mouse mixed glia. **(A)** Lymphocytes were isolated from C57BL/6 mice and mock-treated or treated with 1 μg/ml anti-CD3e and 1 μg/ml anti-CD28 for 1 h. The cells were co-cultured with primary mixed glial cells for 24 h (lymphocytes: glia = 1:0.1), and cell extracts were analyzed by Western blotting using the indicated antibodies. **(B)** Primary glial cells were pretreated with or without the indicated concentration of GA for 1 h, and lymphocytes were mock-treated or treated with 1 μg/ml anti-CD3e and 1 μg/ml anti-CD28 for 1 h. The cells were co-cultured for 24 h. The results shown are representative of three individual experiments.

MS is an inflammatory T-cell-mediated demyelinating disease of CNS that is characterized by activation of immune cells such as lymphocytes and glia^22^,^23^. In an attempt to delineate the molecular mechanism(s) underlying MS, we set out to determine which inflammatory signaling molecules could be affected by an interaction between glia and activated lymphocytes, and then examined the effect of GA on such signaling in CNS immune effector cells. Lymphocytes isolated from C57BL/6 mice were mock-treated or treated with each of anti-CD3e and anti-CD28 for 1 h, and then co-cultured with mouse primary glial cells for 24 h. Using antibodies against representative inflammatory signaling molecules, we explored which signaling molecules could be influenced by the interaction of activated lymphocytes and glia. We found that the levels of tyrosine phosphorylated signal transducer and activator of transcription (STAT) 1 and STAT 3, representative inflammatory molecules, were significantly increased in glial cells co-cultured with activated lymphocytes, but not in those co-cultured with mock-treated control lymphocytes (Fig. 1A(a)). However, tyrosine phosphorylation of STAT1 and −3 were undetectable or low in mono-cultured control lymphocytes or activated lymphocytes (Fig. 1A(b)). Under the same conditions, we did not detect any significant activation of MAPKs in glial cells co-cultured with activated lymphocytes. Next, we examined the effect of GA on the activation of STAT1 and −3 in co-cultured glial cells with activated lymphocytes or mock-treated control lymphocytes. As shown in Fig. 1B, GA treatment considerably reduced the phosphorylation of STAT1 and −3 in glial cells with activated lymphocytes, in comparison with the mock-treated control. Collectively, these results indicate that phosphorylations of STAT1 and −3 are triggered by interaction between activated lymphocytes and glia, and further show that GA significantly reduces this STAT activation.

### GA reduces phosphorylation of STAT1 and −3 by multiple cytokines in glia

**Figure 2:**
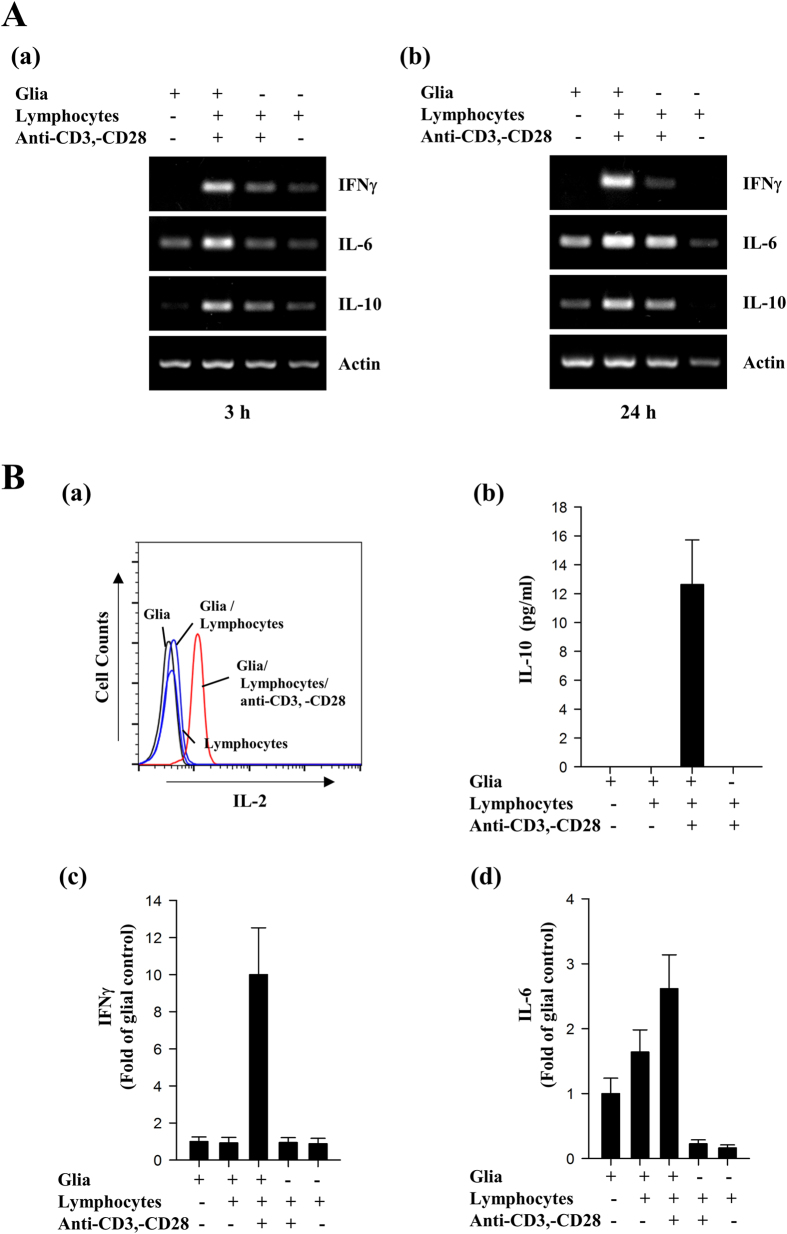
Inflammatory cytokines are markedly increased in co-culture of glia and activated lymphocytes. **(A)** Mouse primary glia were cultured alone or co-cultured with lymphocytes or lymphocytes with 1 μg/ml anti-CD3e and 1 μg/ml anti-CD28 for 3 h (a) or 24 h (b). Transcript levels of the indicated cytokines were determined by RT-PCR analysis. **(B)** The secreted levels of IL-2, IL-10, IFNγ, and IL-6, were measured in the indicated cells using a CBA assay kit. The results shown are representative of three individual experiments.

**Figure 3:**
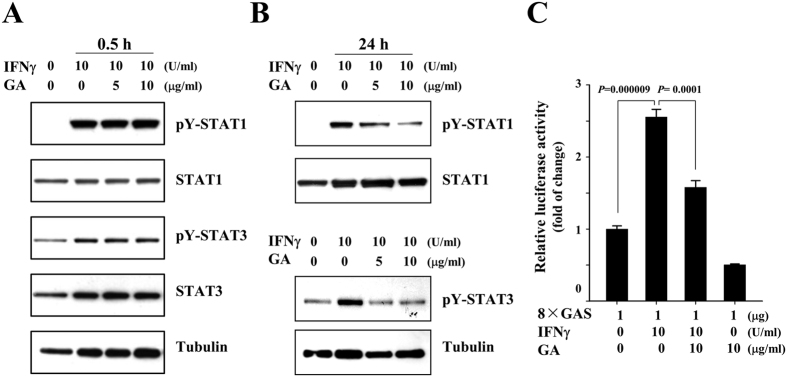
The IFNγ-triggered activations of STAT1 and −3 are attenuated by GA. (**A** and **B**) Rat primary astrocytes were mock-treated or treated with the indicated concentration of GA, and then treated with 10 U/ml IFNγ for 0.5 h or 24 h. Western blot analysis was performed using the indicated antibodies. The data presented are representative of at least three independent experiments. **(C)** Primary astrocytes were transfected with 8 × GAS luciferase reporter plasmids, and then mock-treated or treated with 10 μg/ml GA for 1 h. The cells were mock-treated or treated with 10 U/ml IFNγ for 24 h. Cell extracts were then subjected to a luciferase activity assay. The results shown represent the mean ± SD of triplicate experiments and are representative of three individual experiments.

The above results raised the question of possible mediators that activate phosphorylation of STAT1 and -3 in co-culture of glia and activated lymphocytes. Thus, we examined the levels of several cytokines in glia, lymphocytes, and co-cultured primary glial cells with activated lymphocytes. RT-PCR analysis and cytometric bead array (CBA)-based FACS analysis showed that the levels of several inflammatory cytokines including IFNγ, IL-6, IL-2, and IL-10 were significantly increased in glial cells with activated lymphocytes, compared to those in glia or lymphocytes alone (Fig. 2A and B). In addition, we observed that phosphorylation of STAT1 and -3 was markedly induced by IFNγ or IL-6 in primary glia (Supplementary Figure 1 and Fig. 3A). Together, these results suggest a possibility that activated cell-derived cytokines are responsible for activation of STAT signaling in glia.

IFNγ is a representative activator of tyrosine phosphorylation of STAT1, and a major pro-inflammatory cytokine that is closely associated with MS^24^,^25^,^26^. To validate that GA affects the activation of STAT1 and −3 in glia, we examined its effect on the IFNγ-induced phosphorylations of STAT1 and −3 in primary astrocytes, a major type of glial cell that acts as brain resident immune cells. Primary astrocytes were cultured from the cerebral cortices of 1- to 3-day-old SD rats, and the cells were mock-treated or treated with IFNγ in the presence or absence of 5 or 10 μg/ml GA for 0.5 h, or treated with 5 or 10 μg/ml GA for 1 h, followed by treatment with 10 U/ml IFNγ for 24 h. Western blot analysis showed that IFNγ-triggered phosphorylations of STAT1 and −3 were significantly attenuated by GA at 24 h after treatment with IFNγ. However, we did not detect significant difference between glia treated with IFNγ alone and glia with IFNγ and GA for 0.5 h (Fig. 3A and B). To further demonstrate the effects of GA on activations of STAT1 and −3, we examined the promoter activity of the IFNγ-activated sequence (GAS), which is a representative binding site of STATs. As shown in Fig. 3C, we found that IFNγ-triggered GAS promoter activity was significantly decreased in the presence of GA.

**Figure 4:**
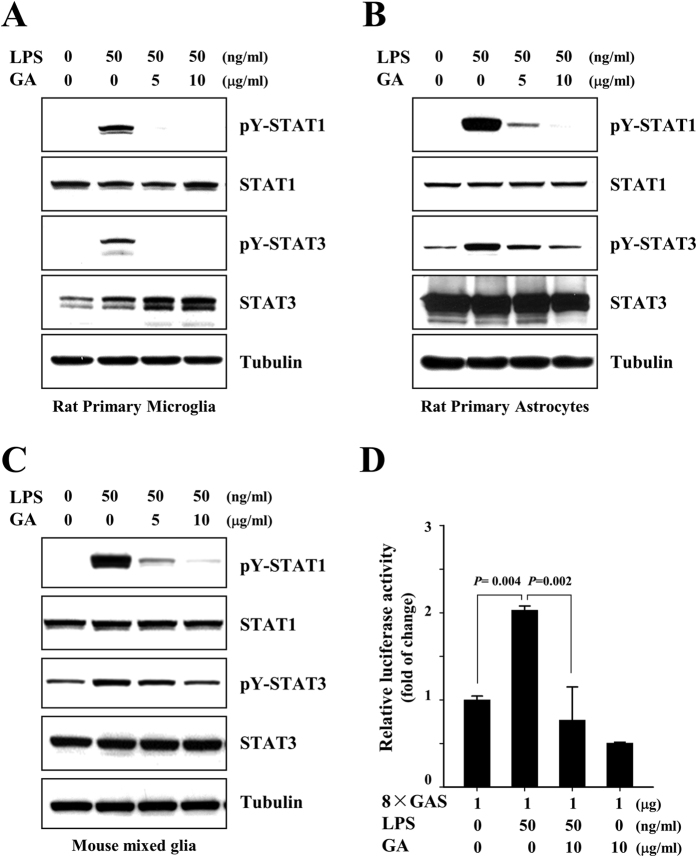
LPS-triggered tyrosine phosphorylations of STAT1 and −3 are reduced by GA in rat primary microglia and astrocytes. **(A)** Rat primary microglia, **(B)** rat primary astrocytes and **(C)** mouse mixed glial cells were mock-treated or treated with 5 or 10 μg/ml GA for 1 h, and then treated with 50 ng/ml LPS for 3 h. Western blot analyses were performed using the indicated antibodies. The data are representative of at least three independent experiments. **(D)** Rat primary astrocytes were transfected with 8 × GAS luciferase constructs, and the cells were mock-treated or treated with 50 ng/ml LPS in the presence or absence of 10 μg/ml GA for 24 h. Cell extracts were then subjected to a luciferase activity assay. The results shown represent the mean ± SD of triplicate samples and are representative of four individual experiments.

Lipopolysaccharide (LPS) has been reported to be a possible environmental trigger of EAE and MS, and also been shown to influence inflammatory demyelinating disease^22^,^25^,^27^,^28^. LPS has been reported to trigger production of inflammatory cytokines, thereby indirectly activating phosphorylation of STAT1 and -3^29^,^30^ To further confirm the effects of GA on activation of STAT1 and −3, we examined the levels of phosphorylated STAT1 and −3 in rat and mouse glia treated with LPS. Consistent with the above results, GA markedly reduced the LPS-triggered tyrosine phosphorylation of STAT1 and −3 in rat primary microglia, rat primary astrocytes, and mouse mixed glial cells (Fig. 4A–C). The LPS-induced up-regulation of 8 × GAS promoter activity was also significantly reduced by GA (Fig. 4D). Taken together, these results provide evidence that GA significantly reduces the activated lymphocyte-, IFNγ- and LPS-induced activations of STAT1 and −3 in glia.

### GA attenuates the STAT1 and −3 signaling in peripheral blood mononuclear cells, but not neuronal cells

**Figure 5:**
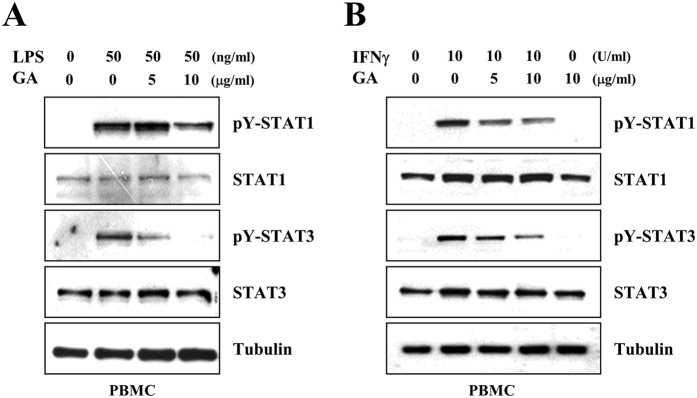
GA shows similar effects on STAT1 and −3 activation in peripheral blood mononuclear cells (PBMCs). PBMCs were isolated from freshly drawn SD rat blood, treated with 5 or 10 μg/ml GA for 1 h, and then either treated with LPS for 3 h **(A)**, or treated with IFNγ for 24 h (**B**). Western blot analyses were performed using the indicated antibodies. The results shown are representative of at least three individual experiments.

To further define the effects of GA in MS, we next examined the effects of GA on the phosphorylations of STAT1 and −3 in peripheral blood mononuclear cells (PBMCs). PBMCs were isolated from SD rat blood, mock-treated or treated with 5 or 10 μg/ml GA for 1 h, and then treated with either 50 ng/ml LPS or 10 U/ml IFNγ for 24 h. Western blot analysis showed that GA markedly reduced the LPS- and IFNγ- induced tyrosine phosphorylations of STAT1 and −3 in rat PBMCs (Fig. 5A,B). These results suggest that GA could inhibit the activations of STAT1 and −3 in PBMCs, in addition to brain resident immune cells.

**Figure 6:**
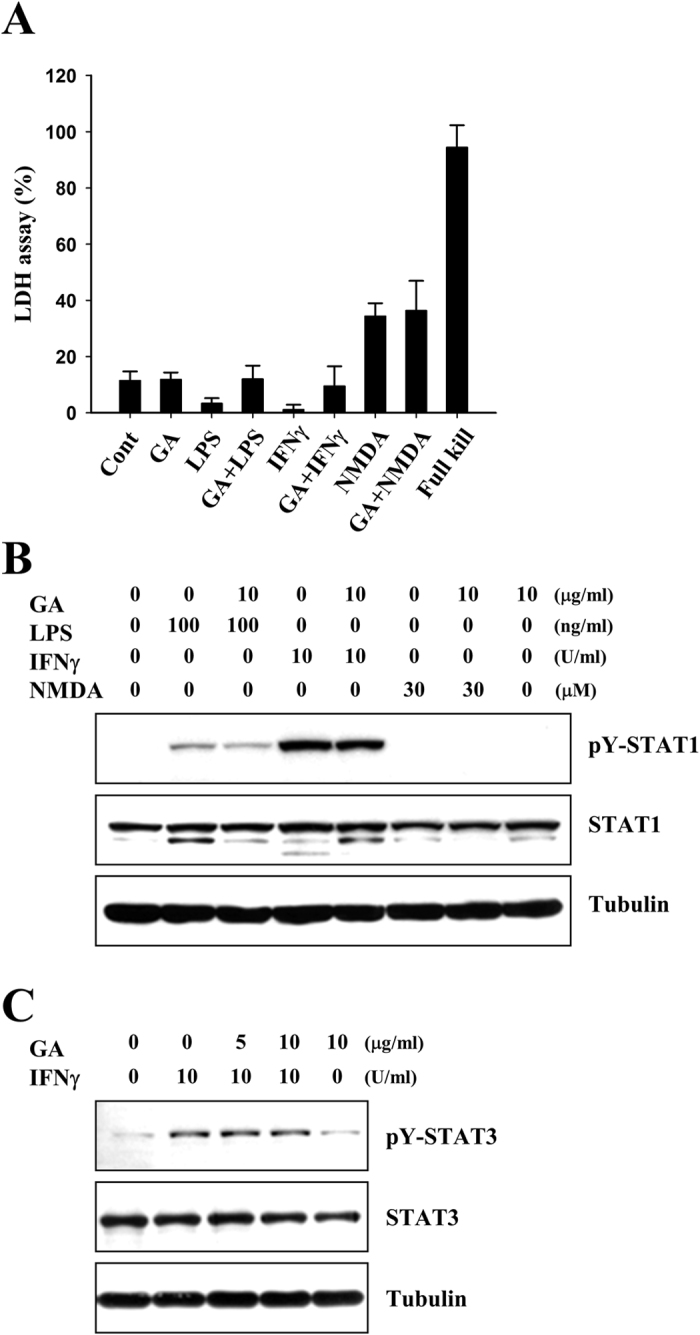
GA does not reduce the activations of STAT1 and −3 in neuronal cells. **(A)** Rat primary cortical neurons were cultured and treated with 100 ng/ml LPS, 10 U/ml IFNγ, or 30 μM NMDA in the presence or absence of 10 μg/ml GA for 72 h. Bars represent the release of LDH from these primary cortical neurons. (**B** and **C**) Primary neuronal cells were mock-treated or treated with 10 μg/ml GA for 1 h, and then treated with 100 ng/ml LPS, 10 U/ml IFNγ, or 30 μM NMDA for 3 h. Western blot analyses were performed using the indicated antibodies. The results shown are representative of at least three individual experiments.

Next, we cultured near-pure cortical neuronal cells from fetal rats, and the cells were mock-treated or treated with 10 μg/ml GA for 1 h, followed by treatment with 100 ng/ml LPS, 10 U/ml IFNγ or 30 μM N-methyl-D-aspartate (NMDA). At 72 h post-treatment, we determined the effects of GA on neuronal cell death using a lactose dehydrogenase (LDH) activity assay. As shown in Fig. 6A, we did not detect any noticeable effects of GA on cortical neuronal cell death. We also failed to observe any change in the phosphorylated levels of STAT1 and −3 by GA in LPS, IFNγ, or NMDA-treated cortical neuronal cells (Fig. 6B and C). These results indicate that GA does not directly affect the activation of STAT1 and −3 in cortical neuronal cells.

### The effects of GA on the phosphorylation of STAT1 and −3 are mediated by SOCS1 and −3, but not IFNγ or IFNγ receptor

**Figure 7:**
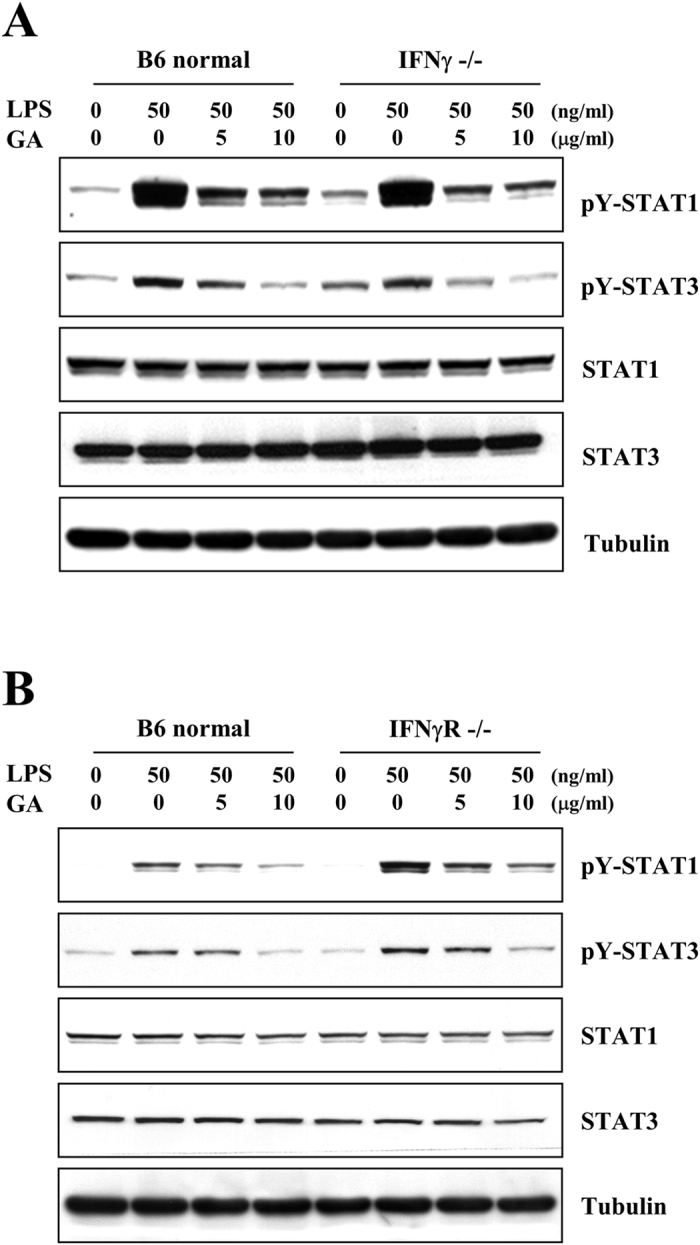
The IFNγ and IFNγ receptor do not appear to mediate the inhibitory effect of GA on the activations of STAT1 and −3. Primary glia from (**A)** IFNγ- or **(B)** IFNγR- deficient mice, were mock-treated or treated with 5 or 10 μg/ml GA for 1 h, and then treated with 50 ng/ml LPS for the 3 h. Western blot analyses were performed using the indicated antibodies. The results shown representative of three individual experiments.

Studies have shown that IFNγ plays a deciding role in whether immune cells attack and injure the CNS, and that LPS stimulates IFNγ production in immune cells^31^,^32^. We thus questioned whether GA could reduce the phosphorylation of STAT1 and −3 via affecting the production of IFNγ or the activation of IFNγ receptor (IFNγR). Using primary glial cells from IFNγ^−/−^ and IFNγR^−/−^ mice, we examined whether deficiencies in IFNγ or IFNγR could affect alter the inhibitory effects of GA on the LPS-triggered phosphorylations of STAT1 and −3. Primary glia were cultured from C57BL/6 (B6), B6.129S7-*Ifng*^tm1Ts^/J or B6.129S7-*Ifngr*^tm1Agt^/J mice. The cells were mock-treated or treated with 5 or 10 μg/ml GA for 1 h, and then mock-treated or treated with 50 ng/ml LPS for 3 h. LPS induced the phosphorylations of STAT1 and −3 in primary glial cells from normal B6 mice. As shown in Fig. 7A, LPS-triggered phosphorylations of STAT1 and −3 were reduced by GA treatment of primary glia from normal and IFNγ^−/−^ mice. GA also showed inhibitory effects on the activation of STAT1 and −3 in IFNγR^−/−^ mice (Fig. 7B). These results indicate that the ability of GA to inhibit the activations of STAT1 and −3 is not mediated through IFNγ^−/−^ or IFNγR^−/−^ system.

**Figure 8:**
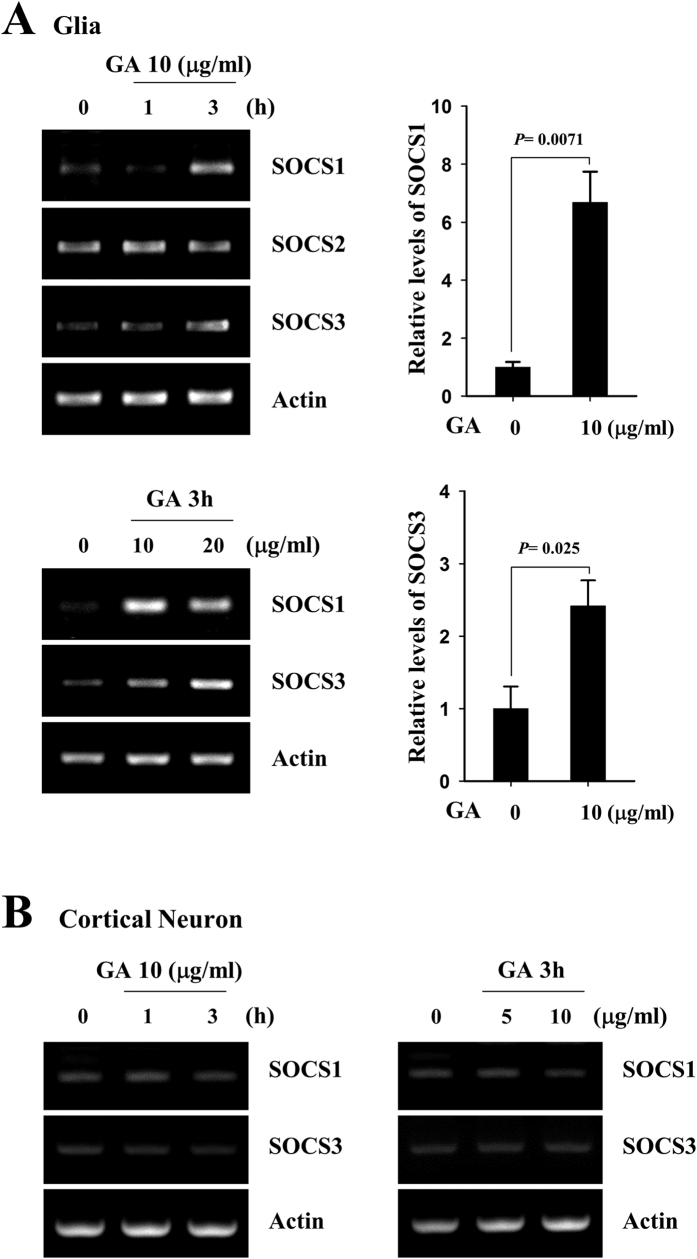
GA elevates the expression levels of SOCS1 and −3 in primary microglia but not in cortical neuronal cells. (**A**) Primary microglia or (**B**) cortical neuronal cells were mock-treated or treated with the indicated concentration of GA for 1 h or 3 h. The transcript levels of SOCS 1 and −3 were measured by RT-PCR analysis, and the results were quantified with the Image J software. The results shown represent the mean ± SD of triplicate experiments and are representative of three individual experiments.

We next examined whether GA could affect the expression of representative inhibitors for STAT phosphorylation. As shown in Fig. 8A, RT-PCR analyses revealed that GA treatment rapidly increased the transcription of the STAT-regulating molecules, suppressors of cytokine signaling (SOCS) 1 and −3 within 3 h. To further investigate the link between SOCS1/3 and effect of GA on phosphorylation of STAT1 and −3, we examined whether GA could induce the expression of SOCS1 and −3 in primary cultured cortical neuronal cells. In accordance with the results from phosphorylation of STAT1 and −3 in neuronal cells, GA did not trigger the transcript level of SOCS1 and −3 (Fig. 8B). These results provide that GA-triggered induction of SOCS1 and −3 may be a negative regulatory mechanism of phosphorylation of STAT1 and −3, thereby affecting activation of immune cells.

### GA decreases production of IL-2 by T lymphocytes co-cultured with glial cells

**Figure 9:**
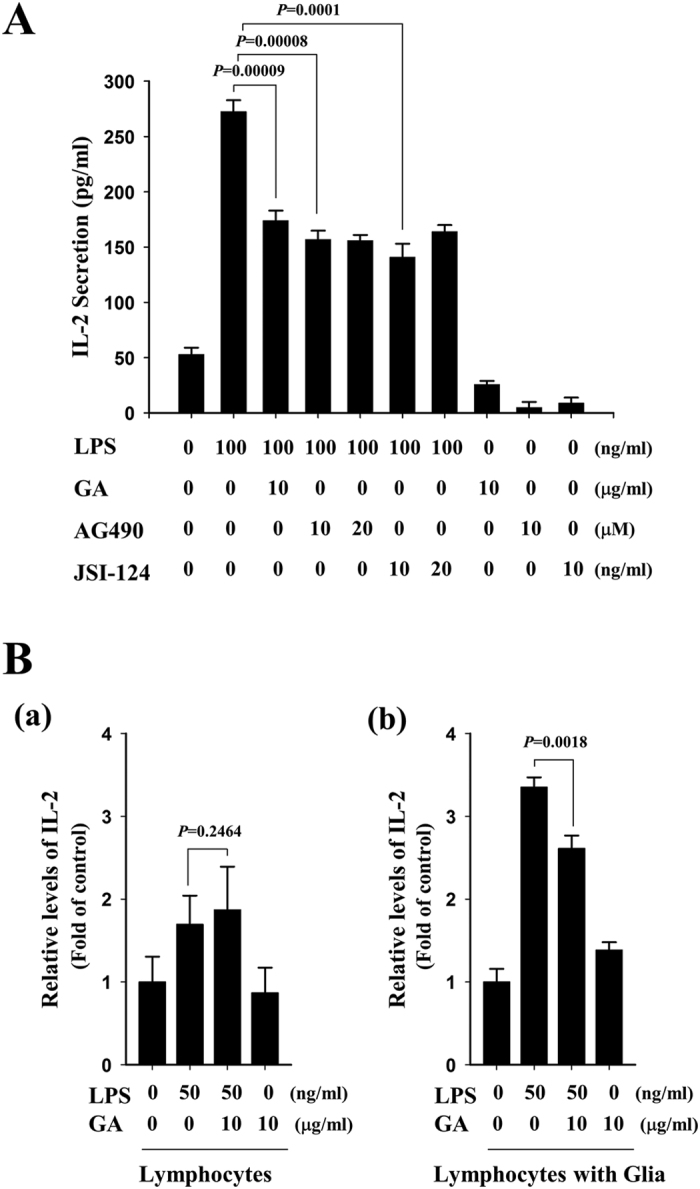
The LPS-triggered production of IL-2 is attenuated by STAT inhibitors as well as by GA. (**A**) Primary glial cells were mock-treated or treated with the indicated concentration of GA, AG490, or JSI-124 for 1 h, and then co-cultured with activated mouse lymphocytes for 24 h (lymphocytes: glia = 1:0.1) in the presence or absence of 100 ng/ml LPS. The levels of IL-2 were measured by ELISA. Graphs are representative of at least three independent experiments, and the data are given as mean ± SD from three different samples. (**B**) Mouse lymphocytes were treated with 0.5 μg/ml anti-CD3e for 1 h, and then cultured without (a) or with (b) mixed glial cells in the presence or absence of GA for 1 h. The cells were then mock-treated or treated with 50 ng/ml LPS for 48 h, and the levels of IL-2 were measured by ELISA.

Upregulation of interleukin-2 (IL-2), a pleiotropic cytokine, has been reported in serum and cerebrospinal fluid (CSF) of patients with clinically active MS^33^, and IL-2-expressing T lymphocytes have been detected in demyelinated plaques in brains of patients with active MS^34^,^35^. We next questioned whether GA-exposed glial cells could indeed influence on T cells, and thus examined the effects of GA on the production of IL-2 in co-cultures containing lymphocytes and glial cells. Mouse lymphocytes were treated with 0.5 μg/ml anti-CD3e, and primary glial cells were mock-treated or treated with 10 μg/ml GA for 1 h. The cells were co-cultured (lymphocytes: glial cells = 1:0.1), and then mock-treated or treated with LPS for 24 h. ELISA analysis was performed to examine the secreted levels of IL-2 in the co-cultured cells. Our results revealed that LPS stimulated the production of IL-2 in co-cultures with mock-treated control glial cells, and the elevated levels of IL-2 were significantly reduced in co-cultures with GA-treated glia (Fig. 9A). We also examined whether GA-dependent reduction of IL-2 was linked with inhibition of STAT signaling using representative inhibitors of STAT signaling. As shown in Fig. 9A, both AG490, a tyrosine kinase inhibitor of JAK2 and JSI-125, a STAT3 inhibitor, showed similar inhibitory effects on the production of IL-2. These results suggest that exposure of glial cells to GA meaningfully reduces the LPS-stimulated activation of the co-cultured cells, and that inhibitors of STAT signaling shows similar effects on the secretion of IL-2 by the co-cultured cells.

To further define the STAT-mediated effects of GA on T cells and glia, we examined whether GA acts directly on lymphocytes versus acting indirectly through glial cells. To do this, we measured the levels of IL-2 in activated lymphocytes alone or activated lymphocytes with primary glia in the presence or absence of GA. Mouse lymphocytes were isolated from lymph nodes and treated with 0.5 μg/ml anti-CD3e. Lymphocytes alone or lymphocytes plus primary glia were treated with GA for 1 h, and then mock-treated or treated with LPS for 24 h. The cells were subjected to ELISA-based measurement of IL-2 levels. LPS significantly stimulated the production of IL-2 in lymphocytes alone and lymphocytes plus glia. LPS-stimulated IL-2 levels were meaningfully reduced in the presence of GA in lymphocytes when co-cultured with glial cells, but this was not detected in lymphocytes alone (Fig. 9B). Taken together, our results show that GA treatment decreases the production of IL-2 by lymphocytes under co-cultured with glial cells, suggesting that GA may act on IL-2 production by lymphocytes through glial cells.

### GA reduces the LPS-triggered production of IL-2 in CD4^+^ T cells co-cultured with glia

**Figure 10:**
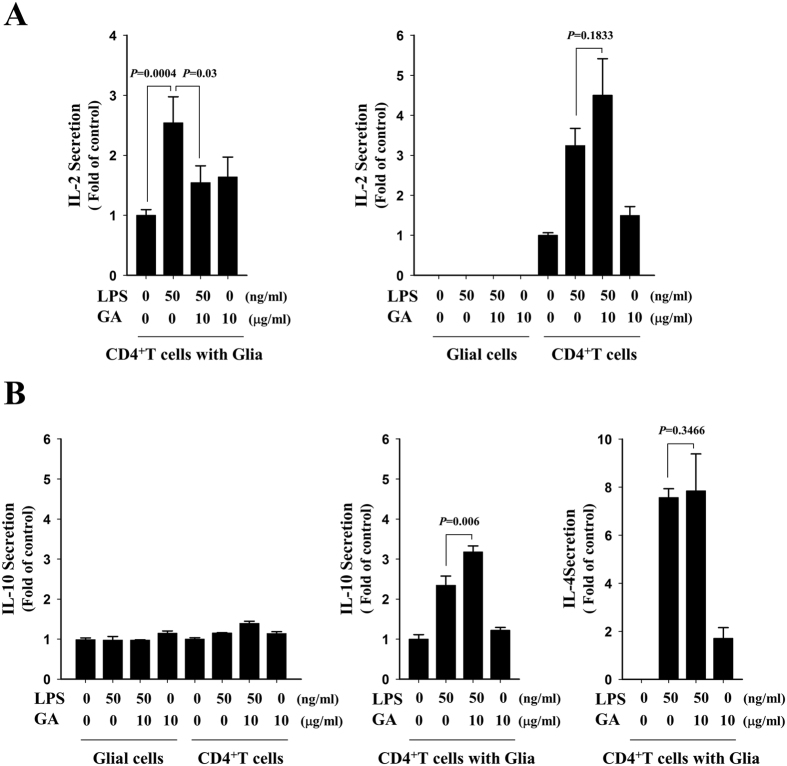
GA reduces the LPS-triggered enhancement of IL-2 production by CD4^+^ T cells co-cultured with primary mixed glial cells. (**A**) Mouse CD4^+^ T cells were isolated from lymph nodes, pre-treated with 0.5 μg/ml anti-CD3e for 1 h, cultured with or without primary mixed glial cells (CD4^+^ T cells: glia = 1:0.1). The cells were mock-treated or treated with GA for 1 h, and then were mock-treated or treated with 50 ng/ml LPS for 24 h. The production of IL-2 by CD4^+^ T cells was measured using CBA-based FACS. (**B**) The levels of IL-10 and IL-4 were measured from the same sample using a CBA assay kit. Graphs are representative of at least three independent experiments, and the data are shown as the mean ± SD from three different samples.

Since CD4^+^ T cells have been known to be the key initiators of tissue destruction and a predominant mediator of neuropathology in MS^1^,^36^,^37^, we isolated CD4^+^ T lymphocytes from B6 mice, and examined the effects of GA on the production of IL-2 by CD4^+^ T cells using cytometric bead array (CBA)-based FACS. CD4^+^ T cells, glial cells, or CD4^+^ T cells plus primary glia were mock-treated or treated with LPS in the presence or absence of GA. As shown in Fig. 10A, IL-2 secretion was increased in LPS-exposed cultures containing CD4^+^ T cells alone or CD4^+^ T cells plus glia, compared to mock-treated control cells. No IL-2 secretion was detected from LPS-treated primary glia. Notably, LPS-triggered induction of IL-2 was significantly reduced by GA in CD4^+^ T cells when cultured with glial cells, but not in CD4^+^ T cells alone (Fig. 10A). Under the same conditions, in contrast, GA did not reduce the levels of IL-10 or IL-4, representative MS-associated Th2 cytokines, in LPS-treated CD4^+^ T cells with glia (Fig. 10B)^38^,^39^,^40^,^41^. These results suggest that GA diminishes the activation of CD4^+^ T cells co-cultured with primary glial cells, but has no such effect on CD4^+^ T cells alone. Collectively, our data indicate that GA appears to affect the interdependent activation of CD4^+^ T cells and glia, and that the regulatory effects of GA on the activity of STAT1 and −3 and the production of IL-2 may be an important molecular mechanism underlying the therapeutic action of GA on MS.

## Discussion

MS is a neuroinflammatory disease that is accompanied by serious disabilities, including motor impairments, fatigue, pain, and cognitive deficits^1^. The brain lesions of MS are characterized by abnormal immune cell infiltrations, demyelination, gliosis, and neuronal degeneration. Increasing clinical and experimental reports support the critical contributions of inflammatory cells, including activated brain-resident immune cells and infiltrating lymphocytes, as key players in the progression of MS. However, the characteristics and interactions were not previously known in detail. In an effort to identify therapeutic targets based on the interactive inflammatory signaling events that occur among the cells of MS lesions, we have sought to decipher the molecular mechanisms underlying the effects of the current disease-modifying therapies for MS. In this study, we show that GA, a globally approved DMD for MS, significantly reduces the activated lymphocytes-triggered phosphorylation of glial STAT 1 and −3, and that this modulates the inflammatory milieu by influencing activation of T cells in the CNS.

The communication between peripheral and CNS immune cells appears to directly or indirectly influence the initiation and regression of MS. Glial cells and infiltrated T cells are located in close proximity to the active lesions of MS patients and EAE models, where they appear to engage in intimate crosstalk^1^,^42^. Accumulating reports indicate that glial cells and T cells can stimulate each other, contributing the pathologic milieu in MS patients and EAE models. In EAE models, IFNγ- and IL-17-producing T cells infiltrate the brain prior to the onset of clinical symptoms, which coincide with the activation of microglia and local production of proinflammatory cytokines^43^. Glia such as microglia and astrocytes play multiple functions in the CNS, including priming, recruiting, and instructing T lymphocytes against neuroinflammatory conditions^44^. They also closely communicate with infiltrating innate immune cells such as neutrophils and mast cells^45^. In this study, we found that phosphorylations of STAT1 and −3 were distinctively enhanced in glial cells that were co-cultured with activated lymphocytes. This suggests that activated lymphocytes may activate glial cells, further enhancing pathological conditions (Fig. 1A). There are also reports that STAT signaling may be a probable target in MS^42^. We thus examined whether GA could affect the phosphorylation of glial STAT1 and −3 by activated lymphocytes. Interestingly, we observed that these phosphorylations of STAT1 and −3 were significantly reduced by GA, but that treatment with this agent did not alter the phosphorylation of ERK, p38, and JNK under the same conditions. GA also markedly reduces the STAT binding promoter activity in both IFNγ- and LPS-treated glial cells. These results suggest that GA can suppress the activation of STAT signaling in glial cells.

Increasing reports indicate that glial cells have multiple functions as brain-resident immune effector cells in the relatively immune-privileged milieu of the CNS. These cells have been implicated in not only immune surveillance, but also in acute and chronic inflammation at all stages of inflammation-associated brain diseases, including MS^1^. To address the molecular mechanism underlying the effect of GA on STAT signaling in glial cells, we explored the effects of GA on STAT-associated signaling events. Neither IFNγ nor IFNγ receptor appears to be linked with the action of GA on STAT signaling. Since GA has been shown to be antigenic and stimulate T cells^6^, we next explored the possibility whether down-regulation of STAT phosphorylation could be due to the GA-triggered glial activation or lymphocyte activation. However, we did not detect significant activation signs of glia or lymphocytes in our experimental conditions (Supplementary Figures 2 and 3), implying that neither effect of GA on glial activation nor effect of GA on lymphocyte activation is the major cause of GA-induced down-regulation of STAT1/3 signaling in co-culture of glia and lymphocytes. Interestingly, we found that expression of suppressor of cytokine signaling (SOCS) 1 and −3, negative regulators of STAT signaling, were significantly increased in GA-treated glia. A previous report showed that a lack of SOCS3 in myeloid lineage cells increased and prolonged JAK/STAT pathway activation compared with that observed in cells from SOCS3 f/f mice^46^. In addition, mice with myeloid-specific deficiency of SOCS3 showed high levels of STAT3 activation and exhibited a severe EAE^47^. The previous reports and our present results therefore suggest that SOCS proteins contribute to MS pathology, and that GA can modulate these functions.

STAT signaling pathway is a pivotal event in immune-associated physiology and pathology, and has received great attention as a therapeutic target^42^. Dysregulation and over-activation of the STAT pathway have been linked to numerous diseases, including MS^48^. Many cytokines such as IFNγ, IL-6, IL-12, IL-23, and GM-CSF have been implicated in the pathogenesis of MS and EAE, which all are reported to activate STAT signaling^42^. In addition, genetic and genome-wide association studies have shown that genes involved in STAT pathway are dysregulated in MS patents^49^. Frishullo *et al*. reported that T cells and monocytes obtained from MS patients during relapse had higher levels of activated STAT3 and lower levels of SOCS3 than comparable cells from patients in remission^42^,^50^. Our present data show that GA treatment specifically suppressed the activation of STAT 1 and −3 in activated glia, and augmented the expression levels of SOCS1 and −3. Furthermore, GA-treated glia showed suppressive effects on the production of IL-2 by CD4^+^ T cells when compared to mock-treated glia. Clinical and experimental studies have shown that GA reduces relapses, new demyelinating lesions, loss of brain tissues, and persistent black holes in patients, and suggested the possibility that GA has a multifaceted mechanism of action^51^. Our results, together with the previous reports, strongly suggest that GA-induced reductions in glial STAT signaling may be a molecular mechanism underlying the action of this therapeutic agent on MS.

**Figure 11:**
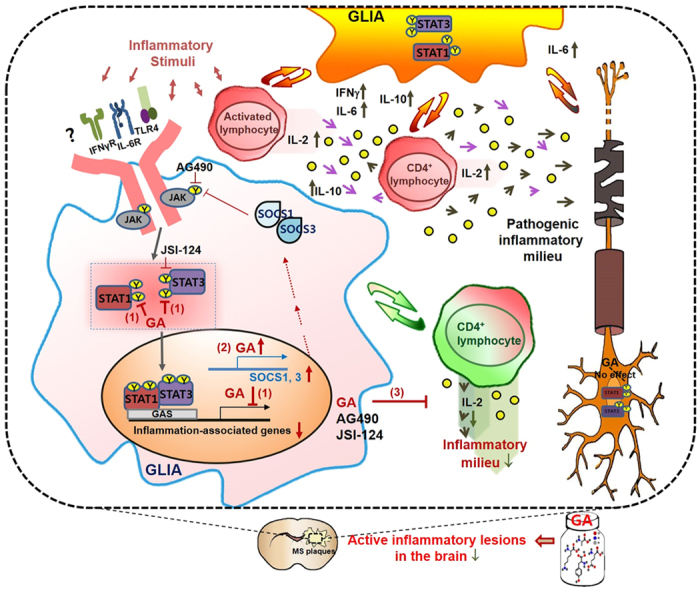
Schematic diagram showing possible actions of GA MS-related brain lesions.

As efforts are being made to develop new treatments for MS, several new agents, including fingolimod, natalizumab, alemtuzumab, and daclizumab, are under study in clinical trials or recently approved^8^,^29^. However, the causes and mechanisms underlying the MS pathophysiology and the action of DMDs still remain to be clarified, which has limited the development of targeted therapeutics with good efficacy and lack of serious adverse effects against this debilitating disease. Our current results show that: (1) activation of glial STAT1 and −3 occurs via a distinctive interaction between brain resident immune cells and infiltrating lymphocytes under neuroinflammatory conditions; (2) this is modulated by GA; and (3) GA significantly suppresses the activation of glial STAT1 and −3, thereby affecting activation of T cells (Fig. 11). These results could, in turn, provide insights into the molecular signatures and pathogenetic mechanisms of MS. Glial cells are the resident immune-effector cells of the CNS. They rapidly respond to pathological stimuli, and have been implicated in the pathophysiology of immune-associated diseases, including MS. Further knowledge of how GA affects brain-resident immune cells and infiltrating immune cells, and how GA modulates STAT-associated immune-responses and neurodegeneration may facilitate the development of more effective therapeutic approaches for progressive MS.

## Methods

### Animals

Six- to eight-week-old C57BL/6 (B6) mice were obtained from Orient Bio (Seongnam, Korea). Sprague-Dawley (SD) rats were purchased from SamTako Bio Korea (Osan, Korea). B6.129S7-*Ifngr*^tm1Agt^/J, and B6.129S7-*Ifng*^tm1Ts^/J mice were purchased from the Jackson Laboratory (Bar Harbor, ME). The mice were maintained under specific pathogen-free conditions in the barrier facility at National Cancer Center. All animal procedures were performed according to the ARRIVE guidelines and National Cancer Center guidelines for the care and use of laboratory animals (AAALAC international 001392). The protocols were reviewed and approved by the Institutional Animal Care and Use Committee (IACUC) of National Cancer Center Research Institute (Approval number; NCC-15–297, NCC-15-298).

### Reagents and antibodies

GA (Copolymer I (P1152)), Minimum Essential Medium Eagle (MEM) and *Salmonella typhimurium* Lipopolysaccharide (LPS) were obtained from Sigma-Aldrich (St. Louis, MO). Dulbecco’s modified eagle’s medium (DMDM) and Fetal Bovine Serum (FBS) were purchased from HyClone (Logan, UT). Rat interferon (IFN) γ was purchased from Calbiochem (La Jolla, CA). Antibodies against phospho-STAT1, total STAT1, phospho-STAT3 and total STAT3 were purchased from Cell Signaling Technology (Danvers, MA). Tubulin antibody was purchased from Sigma-Aldrich (St. Louis, MO).

### Primary microglia, astrocytes, and mixed glial cell cultures

Primary microglia were cultured from the cerebral cortices of 1- to 3-day-old SD rats or B6 mice. Briefly, the tissues were triturated into single cells in MEM containing 10% FBS and plated in 75-cm^2^ T-flasks (0.5 hemisphere/flask SD rats) or 25-cm^2^ T-flasks (2 hemisphere/flask, B6 mice) for 2 weeks. The microglia were detached from the flasks by mild shaking and applied to a nylon mesh to remove astrocytes and cell clumps. Cells were plated in 6-well plates (5 × 10^5^ cells/well), 60-mm^2^ dishes (8 × 10^5^ cells/dish), or 100-mm^2^ dishes (2 × 10^6^ cells/dish) and washed after 1 h later to remove unattached cells before use in experiments. Following removal of the microglia, primary astrocytes were prepared by trypsinization. Cells were demonstrated to consist of >95% authentic microglia and astrocytes based on their characteristic morphology and the presence of the astrocytes marker glial fibrillary acidic protein and the microglial marker CD11b.

### Isolation of rat PBMCs

Peripheral blood mononuclear cells (PBMCs) from freshly drawn SD rat blood were isolated by Ficoll-Paque density gradient centrifugation method (Histopaque-1077, Sigma). Briefly, pooled, heparinized blood was layered on to the Histopaque-1077 (equal volume) in a centrifuge tube and centrifuged at 400 g for 30 min at room temperature (RT). The buffy coat, containing mononuclear cells, was removed carefully. The cells were washed twice with isotonic phosphate buffered saline (PBS) by centrifugation at 400 g for 10 min. The resulting pellet was then re-suspended in MEM medium containing 10% heat-inactivated FBS and penicillin-streptomycin.

### Isolation of mouse lymphocytes and CD4^+^ T cells

Lymphocytes were freshly isolated from peripheral lymph nodes (PLN) (superficial cervical, axillary, brachial and inguinal) and mesenteric LNs. CD4^+^ T cells were obtained using the Magnetic-Activated Cell Sorting (MACS) kit according to the manufacturer’s instruction (Miltenyi Biotec, Bergisch Gladbach, Germany). The magnetically retained CD4^+^ T cells were collected as positively selected cells for further research.

### RT-PCR analysis

Total RNA was isolated using easy-BLUE (iNtRON, Daejeon, Korea) and cDNA was synthesized using avian myeloblastosis virus reverse transcriptase (TaKaRa, Shiga, Japan) according to the manufacturers’ instructions. PCR was performed with 25 cycles of sequential reactions. Oligonucleotide primers were purchased from Bioneer (Daejeon, Korea). The sequences for the PCR primers used are as follows: forward (F) 5′-ATG AGT GCT ACA CGC CGC GTC TTG G-3′ (R) 5′-GAG TTC ATT GAC AGC TTT GTG CTG G-3′ for IFNγ; (F) 5′-CAC GAT TTC CCA GAG AAC ATG TG-3′ (R) 5′-ACA ACC ACG GCC TTC CCT ACT T-3′ for IL-6; (F) 5′-ACC TGC TCC ACT GCC TTG CT-3′ (R) 5′-GGT TGC CA AGC CTT ATC GGA-3′ for IL-10; (F) 5′-AAG ACG TCA GCT GGA CCG AC-3′ for suppressor of cytokine signaling 1 (SOCS1); (F) 5′-AAG ACG TCA GCT GGA CCG AC-3′ (R) 5′-TCT TGT TGG TAA AGG CAG TCC C-3′ for SOCS2; (F) 5′-ACC AGC GCC ACT TCT TCA CG-3′ (R) 5′-GTG GAG CAT CAT ACT GAT CC-3′ for SOCS3; (F) 5′-CAT GTT TGA GAC CTT CAA CAC CCC-3′ and (R) 5′-GCC ATC TCC TGC TCG AAG TCT AG-3′ for actin.

### Luciferase assay

Transient transfections were performed in triplicate on 35 mm dishes using Lipofectamine 2000 reagents as instructed by the manufacturer (Invitrogen Life Technologies, Carlsbad, CA). Luciferase assay was performed according to the manufacturer’s instruction (Promega, Madison, WI). The light intensity was measured for 30 s on a Victor^TM^ Light 1420 Luminescence Counter (Perkin Elmer, Waltham, MA). Luciferase activity was normalized by measurement of the β-galactosidase activity.

### Western blot analysis

Cell lysate was centrifuged for 20 min at 13,000 rpm at 4 °C, and supernatant proteins were separated by SDS-PAGE on 10% gels and transferred to nitrocellulose membranes. The membranes were incubated with primary antibodies and horseradish peroxidase–conjugated secondary antibodies and then visualized using an enhanced chemiluminescence system.

### ELISA

ELISA kits were used according to the manufacturers’ protocols. After treatment with stimuli, 100 μl of conditioned media was collected and assayed using ELISA kits for mouse IL-2 (ebioscience, San Diego, CA), according to the manufacturers’ procedures.

### Cytokine analysis using cytometric bead array (CBA)

Cytokine levels were determined using CBA. The procedure was carried out according to the manufacturer’s instructions (BD Biosciences, San Jose, CA). Briefly, 10 μl of each mouse capture bead suspensions were mixed for 50 μl of each supernatants sample, and the mixed bead suspensions were transferred to each assay tube. Standard dilutions or test samples were added to the appropriate tubes (50 μl/tube), and PE detection reagent (50 μl) was added. After 2 h incubation in dark at RT, samples were washed and were performed using a Becton Dickinson FACSCalibur (BD Biosciences) system. Data were analyzed using FlowJo software (Treestar, Inc, San Carlos, CA).

### Cortical neuronal cultures

Cortical neuronal cultures were prepared from fetal rat at 17 days of gestation as described previously^52^. Briefly, dissociated cortical cells were plated onto poly-D-lysine/laminin-coated plates (Nunc, Rochester, NY, USA) at 75 000 cells/cm^2^ in DMEM with 20 mmol glucose, 2 mmol glutamine, and 5% FBS. This procedure routinely produced near-pure cortical neuronal cultures through days *in vitro* (DIV) 10 to DIV14 consisting of >96% neurons, <1% astrocytes, and <0.5% microglia.

### Lactate dehydrogenase assay

The lactate dehydrogenase (LDH) level was determined by measuring absorbance at a wavelength of 340 nm in kinetics mode for 5 minutes on a microplate reader (Molecular Devices). The percentage cytotoxicity was calculated as follows using total cellular LDH as a low control: Cytotoxicity (%) = [(Experimental Value-Low Control)/(High Control-Low Control)] ×100%.

### Data analysis

All data were expressed as the mean 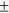 SD and all statistical analyses were performed using the SigmaPlot software version 12.0 (Systat Software Inc.). Results were considered statistically significant when *p* < 0.05.

## Additional Information

**How to cite this article**: Ahn, Y.-H. *et al*. Glatiramer acetate attenuates the activation of CD4^+^ T cells by modulating STAT1 and −3 signaling in glia. *Sci. Rep.* **7**, 40484; doi: 10.1038/srep40484 (2017).

**Publisher's note:** Springer Nature remains neutral with regard to jurisdictional claims in published maps and institutional affiliations.

## Supplementary Material

Supplementary Information
