# Supplementary material for: Glatiramer acetate attenuates the activation of CD4+ T cells by modulating STAT1 and −3 signaling in glia
Source: Sci Rep. 2017 Jan 17;7:40484. doi: 10.1038/srep40484 (PMC5240344; doi:10.1038/srep40484)
Supplement: Supplementary Information [file srep40484-s1.pdf]

Supplementary Information

Glatiramer acetate attenuates the activation of $CD4^+$ T cells by modulating STAT1 and -3 signaling in glia

Ye-Hyeon Ahn[1,5], Sae-Bom Jeon[1], Chi Young Chang[1], Eun-Ah Goh[2], Sang Soo Kim[3], Ho Jin Kim[2,4], Jaewhan Song[5], Eun Jung Park[1,2]*

[1]Cancer Immunology Branch, National Cancer Center, Goyang, South Korea,

[2]Dept. of System Cancer Science, Graduate School of Cancer Science and Policy, Goyang, South Korea,

[3]Radiation Medicine Branch, National Cancer Center, Goyang, South Korea,

[4]Dept. of Neurology, National Cancer Center, Goyang, South Korea,

[5]Dept.of Biochemistry, College of Life Science and Biotechnology, Yonsei University, Seoul, Korea.

- Corresponding author: Eun Jung Park, Ph.D. Chief of Cancer Immunology Branch, Research Institute, Professor of Cancer System Science, Graduate School of Cancer Science and Policy, National Cancer Center, Goyang, 410-769, Korea. Tel: 82-31-920-2530, 2543 Fax: 82-31-920-2542 .

Supplementary Figure 1

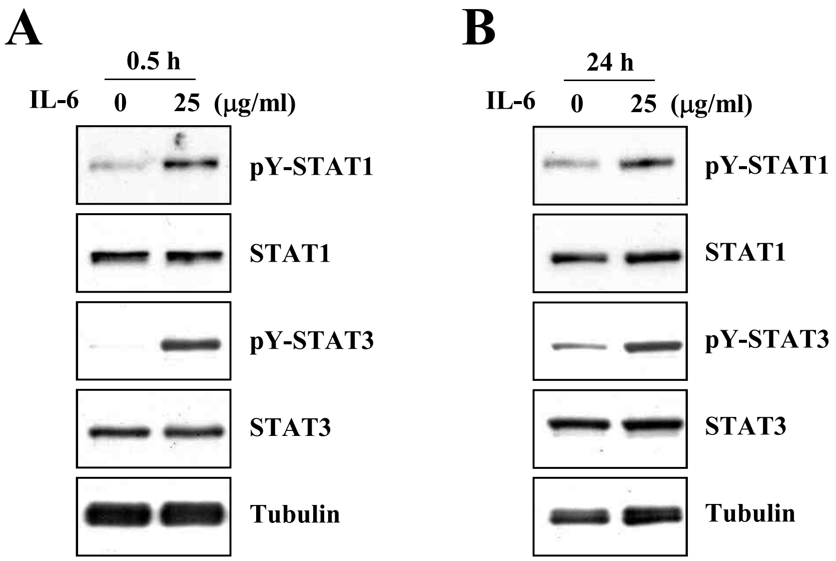

Supplementary Figure 1. Mouse primary glia were mock-treated or treated with 25 μg/ml IL-6 for **(A)** 0.5 h or **(B)** 24 h. Total extracts were subjected to Western blot analyses. The membrane was sequentially probed with the indicated antibodies.

Supplementary Figure 2

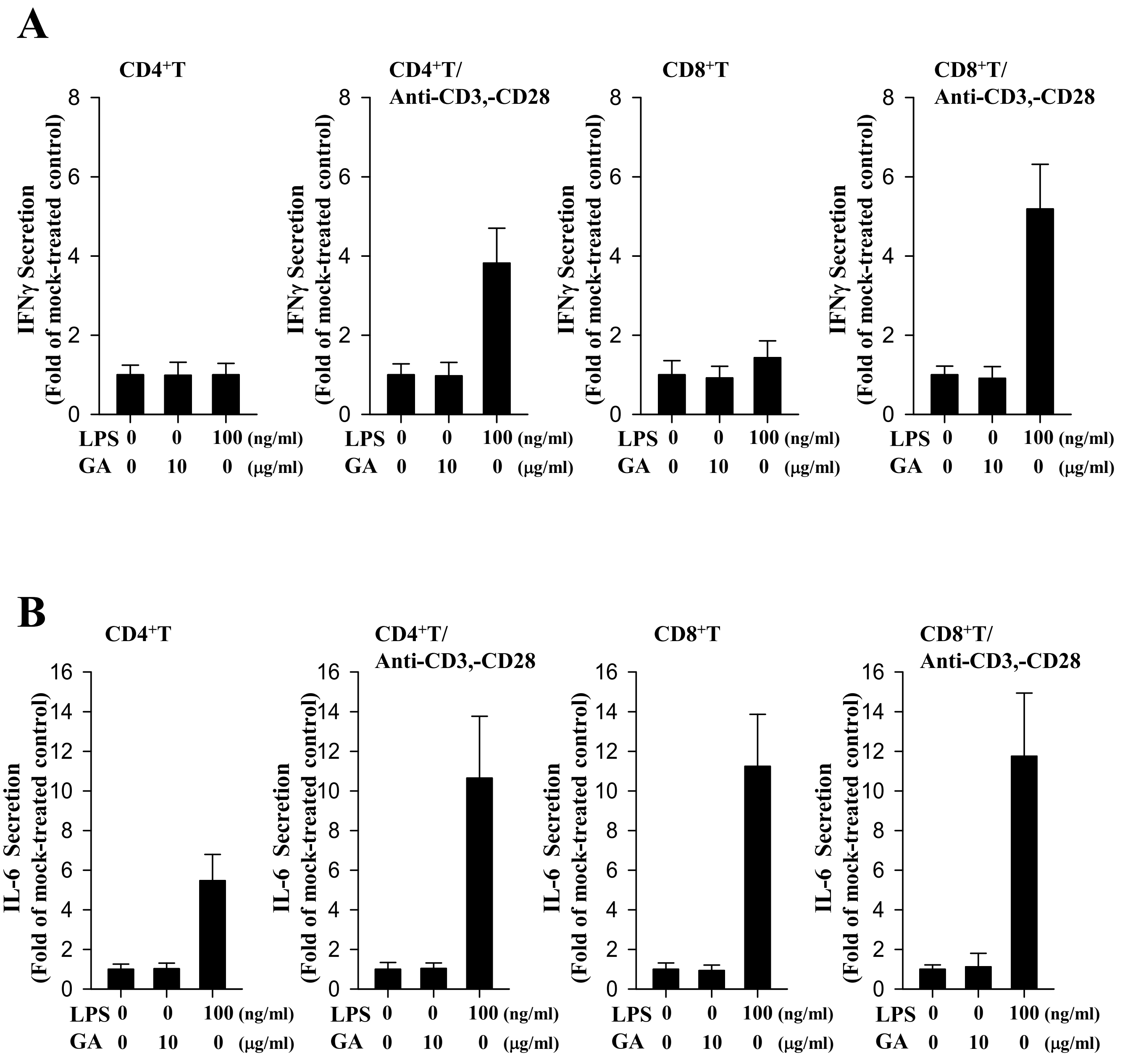

Supplementary Figure 2 continued

C

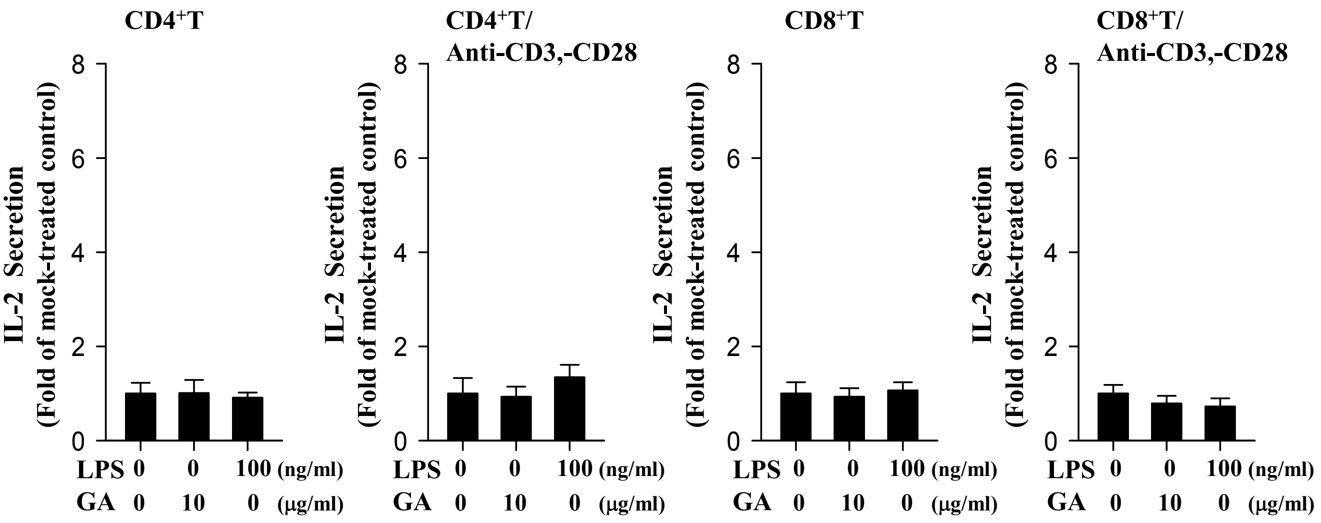

Supplementary Figure 2. Mouse CD4$^+$ T cells or CD8$^+$ T cells were isolated from lymph nodes of C57BL/6 mice, pre-treated with 1 μg/ml anti-CD3e and 1 μg/ml anti-CD28 for 1 h or without. Each cells were mock-treated or treated with GA or LPS as a positive control for 24 h. The levels of **(A)** IFNγ, **(B)** IL-6, and **(C)** IL-2 secreted by CD4$^+$ or CD8$^+$ T cells were measured using a CBA-based assay.

Supplementary Figure 3

A **Rat primary microglia (24h)**

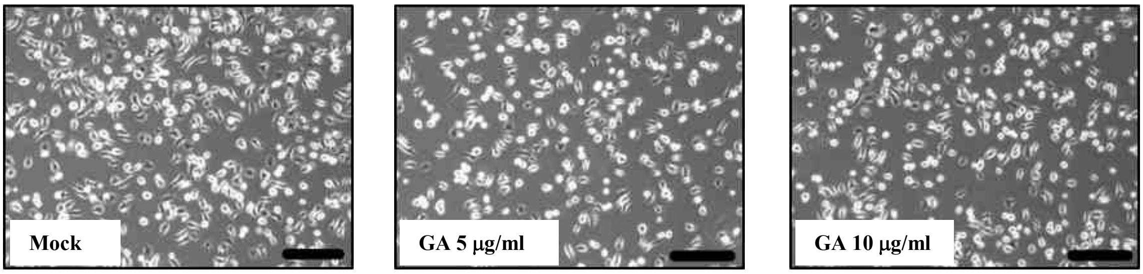

Scale bar; 100 μm

B **Mouse mixed glia (24h)**

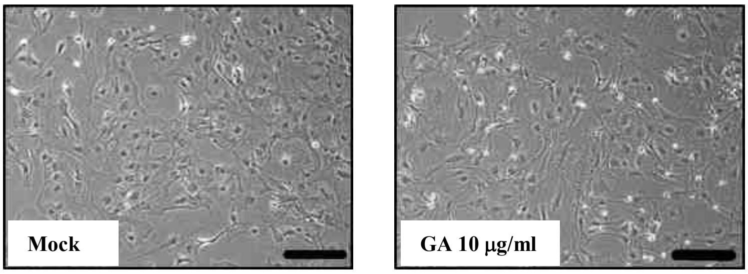

Scale bar; 100 μm

Supplementary Figure 3. (A) Rat primary microglia or **(B)** mouse mixed glia were mock-treated or treated with 5 or 10 μg/ml GA for 24 h. Cell morphology was observed using Leica DMIL inverted microscope (×100)
